# Inference comprehension in text reading: Performance of individuals with right- versus left-hemisphere lesions and the influence of cognitive functions

**DOI:** 10.1371/journal.pone.0197195

**Published:** 2018-05-24

**Authors:** Marcela Lima Silagi, Marcia Radanovic, Adriana Bastos Conforto, Lucia Iracema Zanotto Mendonça, Leticia Lessa Mansur

**Affiliations:** 1 Department of Physical Therapy, Speech-Language Pathology and Audiology, and Occupational Therapy, School of Medicine, Universidade de Sao Paulo, Sao Paulo, Brazil; 2 Department of Psychiatry, School of Medicine, Universidade de Sao Paulo, Sao Paulo, Brazil; 3 Department of Neurology, School of Medicine, Universidade de Sao Paulo, Sao Paulo, Brazil; Universita degli Studi di Udine, ITALY

## Abstract

**Background:**

Right-hemisphere lesions (RHL) may impair inference comprehension. However, comparative studies between left-hemisphere lesions (LHL) and RHL are rare, especially regarding reading comprehension. Moreover, further knowledge of the influence of cognition on inferential processing in this task is needed.

**Objectives:**

To compare the performance of patients with RHL and LHL on an inference reading comprehension task. We also aimed to analyze the effects of lesion site and to verify correlations between cognitive functions and performance on the task.

**Methods:**

Seventy-five subjects were equally divided into the groups RHL, LHL, and control group (CG). The Implicit Management Test was used to evaluate inference comprehension. In this test, subjects read short written passages and subsequently answer five types of questions (*explicit*, *logical*, *distractor*, *pragmatic*, and *other*), which require different types of inferential reasoning. The cognitive functional domains of attention, memory, executive functions, language, and visuospatial abilities were assessed using the Cognitive Linguistic Quick Test (CLQT).

**Results:**

The LHL and RHL groups presented difficulties in inferential comprehension in comparison with the CG. However, the RHL group presented lower scores than the LHL group on *logical*, *pragmatic* and *other* questions. A covariance analysis did not show any effect of lesion site within the hemispheres. Overall, all cognitive domains were correlated with all the types of questions from the inference test (especially *logical*, *pragmatic*, and *other*). Attention and visuospatial abilities affected the scores of both the RHL and LHL groups, and only memory influenced the performance of the RHL group.

**Conclusions:**

Lesions in either hemisphere may cause difficulties in making inferences during reading. However, processing more complex inferences was more difficult for patients with RHL than for those with LHL, which suggests that the right hemisphere plays an important role in tasks with higher comprehension demands. Cognition influences inferential processing during reading in brain-injured subjects.

## Introduction

Text reading is a complex task because it demands the integrity of linguistic competencies that support the decoding and comprehension processes [1]. Text comprehension requires not only the processing of single words and sentences but also the integration of sentences to obtain coherence. The processes needed to achieve coherence include a dynamic balance between the mental representations formed throughout reading and other strategies, such as the elaboration of inferences [2].

Inferences are defined as mental representations that the listener/reader builds based on the application of the subject’s own knowledge along with the explicit information in the text, making it possible to establish relations and associations for the comprehension of implicit information [3]. To perform inferential processing, the individual must be able to analyze beyond the provided information, using associations based on world knowledge, as well as assumptions and deductions about a specific subject, other contextual factors, and resources from the text itself [4].

The literature indicates that individuals with right-hemisphere lesions (RHL) have difficulties with inferential processing compared to cognitively healthy individuals [5–8]. However, comparative studies of the performance of individuals with RHL and left-hemisphere lesions (LHL) are scarce, especially regarding written comprehension. Compared to patients with LHL, those with RHL present additional difficulties in contexts related to the perception of emotion and characters’ motivation, as well as in more ambiguous and incomplete scenarios [9–11]. Hence, comparative studies indicate that lesions in both hemispheres hinder inferential comprehension, depending on the type and complexity of the stimulus.

In addition, studies carried out with healthy individuals using neuroimaging techniques have demonstrated bihemispheric coactivation during inferential processing, with involvement of the RH in more complex challenges such as comprehension of less familiar scenarios [12], ambiguous concepts [13], contents with weaker semantic relations [14], contexts with less consistent information [15], conversation [16] and comprehension of global coherence [17].

In relation to the specific brain areas involved in inferential processing, several studies have confirmed the participation of a broad neural network in the LH and additional homologous areas of the RH (especially the frontal and temporal lobes) in the comprehension of complex material [18–25]. Some of these results have highlighted the prominent activation of the frontal lobe during the generation of inferences [18, 22, 24], but the influence of different areas of each hemisphere in brain-injured subjects is less clear.

Similarly, the role of other cognitive domains in inferential comprehension is also little understood. It is assumed that inferential processing imposes a high cognitive demand [26]. Studies have shown active participation of working memory in inferential processing in healthy individuals [14, 19, 27], as well as in brain-damaged subjects [10]. However, the relationship between inferential ability and other cognitive functions must be further studied, and this is one of the distinctive features of our study.

The aims of this study were as follows: 1) To compare the performance of patients with RHL and LHL, matched with a control group, on an inferential reading comprehension task; 2) To analyze the influence of lesion site (anterior, posterior, and anteroposterior) on this ability; and 3) To verify the correlation between cognitive functions (attention, memory, language, executive functions, and visuospatial abilities) and the performance of each group on the inferential task.

Based on findings reported in the literature, our hypotheses were as follows: 1) Lesions in both hemispheres would lead to difficulties in making different types of inferences, with patients with RHL presenting greater difficulty than those with LHL in complex inferences, 2) Patients with anterior lesions would present greater difficulty than those with posterior lesions in making inferences, and 3) Attention, memory, and executive functions would correlate strongly with the inferential process in all modalities except the *explicit* type.

## Materials and methods

This study was approved by the Ethics Committee for the Analysis of Research Projects (CAPPesq) of the Hospital das Clínicas, School of Medicine, Universidade de São Paulo, under protocol number CAAE 12115. All subjects signed the Free and Informed Consent to participate in the study.

### Sample

The sample was composed of 75 subjects divided into three groups: RHL = 25 subjects with right-hemisphere lesions, LHL = 25 subjects with left-hemisphere lesions, and control group (CG) = 25 cognitively healthy subjects. The groups were matched in terms of sex, age and level of education.

Subjects with brain injury were recruited from a Speech-Language Pathology and Audiology ambulatory service for patients with neurological disorders at a university-linked hospital. The subjects recruited for the CG were volunteers from the community living in the same city.

Subjects were required to meet the following criteria to participate in the study: age greater than or equal to 18 years, four or more years of education, right-hand dominance (determined by the Edinburgh Inventory) [28], and Brazilian Portuguese as a native language.

Subjects with brain injury (groups RHL and LHL) were required to present chronic unilateral (LH or RH) lesions (at least six months after injury—one patient in the RHL group was admitted four months after onset after clinical judgment by the neurological and speech therapist teams, considering that his performance was stable and resembled those of chronic patients) of ischemic vascular etiology in the middle cerebral artery region, documented by neuroimaging (computerized tomography or magnetic resonance imaging). These subjects could present mild to moderate language and communication deficits, but they had to be able to decode written language. Therefore, the subjects were submitted to the Boston Diagnostic Aphasia Examination (BDAE) (short form) [29] and to the Montreal Communication Evaluation Battery [30].

The exclusion criteria for the RHL and LHL groups were as follows: clinical diseases not under adequate control; recent vascular brain injury or lesions located in the brainstem, cerebellum or areas supplied by the anterior or posterior cerebral arteries; presence of other neurological and/or psychiatric diseases; presence of linguistic-cognitive impairments that would hinder the comprehension of the tasks and the ability to decode and understand simple sentences; crossed aphasia; left-hand dominance or cross-dominance; and poor, uncorrected visual acuity.

Subjects in the CG were required to meet the health criteria described in the Mayo Older American Normative Studies (MOANS) [31]. Additionally, they were required to obtain scores compatible with the normal range for the Brazilian population on the Mini-Mental State Examination (MMSE) cognitive screening test [32, 33] and the 21-item Hamilton Depression Rating Scale [34, 35]. Finally, in order to be in the CG, subjects were required to demonstrate their visual function and ability to understand simple written paragraphs, as evaluated by the Reading Comprehension—Sentences and Paragraphs subtest of the BDAE (shortened version) [29].

### Instruments

The instrument used for evaluating inferential comprehension in reading was the Implicit Management Test, translated from the original French version (*La Gestion De L´Implicite*) [36]. Standardization and adaptation for the Brazilian population was carried out with 224 healthy subjects, classified according to age (young adults, adults and elderly) and level of education (low, medium, and high) [37].

The test is designed to evaluate adult subjects with cognitive and communicative complaints. The assessment comprises 20 short text passages in the form of stories. Each text consists of a statement with two, three or four propositions, with dialogues between two speakers or the description of a verbal interaction. The statements are composed by affirmative propositions that describe a fact or expose a problem situation. The texts contain explicit and implicit information, necessary for the correct interpretation during reading. Subjects must read (out loud or silently) each text and answer three questions using “yes”, “no” or “I cannot answer”. Questions are classified into five types (*explicit*, *logical*, *pragmatic*, *other* and *distractor*), according to the inferential reasoning required, as shown in Table 1.

**Table 1:** Types of questions from the Implicit Management Test.

Type of question	Explanation	Example
Explicit (11 questions)	Require paraphrases or literal translation of the statement.	Nadia called Lucas and told him: “My goodness, have you seen the time?”, and Lucas answered: “Yes, I know, but I can’t find my car keys.” *Has Lucas lost the keys to his car?*
Logical (12 questions)	Engage the use of formal reasoning and processes of deduction. Admit only one answer and do not accept divergent arguments.	My neighbor’s cat never meows, except when it hasn’t eaten for a long time. Today, I heard the cat meowing all morning. *Did my neighbor feed her cat this morning?*
Pragmatic (18 questions)	Require knowledge of usual scripts, logical and coherent action plans and conformance to discursive rules.	After the weather report, Brigitte said to herself: “I mustn’t forget my umbrella tomorrow”. *Does Brigitte like getting wet?*
Other (6 questions)	Require handling of logic operations together with pertinent contextualization (combination of logic and pragmatic competencies).	Peter says: “It costs a lot of money to go to Canada; I can’t go there right now”. *Does Peter have a lot of money right now?*
Distractor (13 questions)	The subject should answer with “Cannot answer” because the information requested does not exist in the test, explicitly or implicitly. The questions were asked in order to guide the subjects to deviate from an interpretive approach and give an explanation that they would not have considered spontaneously.	Rose says to Suzanne: “Stop eating or you’ll put on weight!” and Suzanne replies: “So what, men like it”. *Is Rose married?*

The instrument used to evaluate cognitive functions was the Cognitive Linguistic Quick Test (CLQT) [38], composed of the subtests “Personal Facts”, “Symbol Cancellation”, “Confrontation Naming”, “Clock Drawing”, “Story Retelling”, “Symbol Trails”, “Generative Naming”, “Design Memory”, “Mazes” and “Design Generation”. The score in each task contributes to the severity rating for one or more cognitive domains, with different weights attributed to each subtest. The following cognitive domains were evaluated: attention, memory, language, executive functions and visuospatial skills. The score for each domain was correlated with the performance on the inferential comprehension test.

The evaluation was carried out individually in a quiet room in two two-hour sessions. The administration of the tests followed the instructions in the original manuals. In each test, one point was given for each correct answer. Therefore, the higher the score, the better the performance.

### Statistical analysis

For the descriptive analysis, the mean, standard deviation and variance were calculated for each of the demographic variables, performance on the Implicit Management Test, and the CLQT tasks for the three groups.

The means for continuous data were compared using the Kruskal-Wallis test (considering the non-Gaussian distribution of the data), except for the age variable (one-way ANOVA due to the Gaussian distribution of the data), and all significance values were subjected to the Bonferroni correction. Subgroup distribution regarding sex was compared using Pearson’s chi-squared test, and the mean time since injury was compared using the Mann-Whitney U test.

To analyze the possible influence of lesion site on Implicit Management Test performance, we conducted a general linear model (GLM) analysis using performance on the different types of questions as the dependent variables and lesion sites as covariates. The lesion sites were classified as a) anterior (frontal), b) posterior (temporal, parietal and parietotemporal) or c) anteroposterior (frontotemporal, frontoparietal and frontoparietotemporal). We also performed a more thorough analysis by subdividing the RHL and LHL groups according to anterior (frontal) *versus* posterior (temporal, parietal, or parietotemporal) lesions and comparing the four subgroups.

The association between scores obtained on the Implicit Management Test and performance on the CLQT for the cognitive functions of attention, memory, language, executive functions and visuospatial abilities was tested in the general sample and the three groups using the Spearman’s rank-order correlation.

A statistical significance level of 0.01 was adopted for all analyses after applying the Bonferroni correction for multiple comparisons.

## Results

Table 2 shows the demographic and clinical data of the sample.

**Table 2:** Demographic and clinical characteristics of the sample.

Variable	CG	LHL	RHL	P	Intergroup comparison
	M (SD)	M (SD)	M (SD)		
	Min-Max	Min-Max	Min-Max		
Age*	54.4 (9.8)	54.1 (13.0)	52.4 (10.4)	0.667	NS
	28–76	20–70	33–74		
Level of education	10.6 (3.4)	10.8 (4.5)	10.6 (4.9)	0.194	NS
	4–18	4–24	4–23		
Sex^#^				0.687	NS
- Male	10	13	12		
- Female	15	12	13		
Lesion site					
- Anterior	NA	7	6	0.446	NS
- Posterior		13	10		
- Antero-posterior		5	9		
Time since injury (months)ǂ	NA	20.1 (14.0)	22.0 (25.4)	0.255	NS
		6–72	4–120		

Kruskal-Wallis test;

*ANOVA with Bonferroni correction;

^#^Pearson chi-square test;

^ǂ^ Mann-Whitney U test

NS = not significant

No statistically significant differences were found regarding age, education, sex, site or time since brain injury.

Table 3 shows the performance of the sample on the Implicit Management Test.

**Table 3:** Performance of the groups on the Implicit Management Test.

Type of question	CG	LHL	RHL	p	Intergroup comparison
	M (SD)	M (SD)	M (SD)		
	Min-Max	Min-Max	Min-Max		
Explicit	10.1 (0.8)	9.1 (1.2)	9.1 (1.5)	0.014	NA
	9–11	6–11	7–11		
Logical	9.7 (1.3)	8.1 (2.2)	6.6 (2.4)	<0.0001	All differ
	7–12	4–12	2–12		
Pragmatic	14.8 (1.7)	13.1 (1.4)	10.8 (3.0)	<0.0001	All differ
	11–18	10–15	3–15		
Other	4.6 (0.7)	3.7 (1.0)	2.6 (1.5)	<0.0001	All differ
	4–6	1–5	0–6		
Distractor	10.4 (1.5)	8.2 (3.3)	7.7 (4.0)	0.028	NA
	8–13	1–13	0–13		
Total	49.6 (3.8)	42.5 (6.0)	36.9 (8.9)	<0.0001	All differ
	43–57	32–54	15–51		

Kruskal-Wallis test. Statistical significance was defined as p < 0.01. NA = not applicable.

The CG, RHL and LHL groups differed in performance on *logical*, *pragmatic* and *other* questions, as well as total score. The RHL group presented the lowest scores of any group in all types of inferences and in total score.

Analysis of covariance did not show any influence of lesion site (anterior, posterior, or anteroposterior) on inferential comprehension ability (p = 0.369) in the overall sample of brain-damaged subjects. We then performed a more thorough analysis by further subdividing the RHL and LHL groups according to anterior (frontal) *versus* posterior (temporal, parietal, or parietotemporal) lesions. The comparison among the four subgroups (anterior LHL/posterior LHL/anterior RHL/posterior RHL) revealed that the site of the lesion did not influence the performance of subjects in terms of *explicit* (p = 0.164), *logical* (p = 0.310), *pragmatic* (p = 0.714), *distractor* (p = 0.702), or total scores (p = 0.227) in the RHL or LHL group. However, we did encounter a trend-level difference in performance for *other* questions (p = 0.051), where patients with right frontal lesions performed worse than patients with right posterior or left (anterior or posterior) lesions.

Table 4 shows the correlations between the cognitive domains assessed by the CLQT and performance on the Implicit Management Test for the overall sample.

**Table 4:** Correlations between types of inferences and cognitive domains in the overall sample.

Type of question	Cognitive functions				
	Attention	Memory	Executive functions	Language	Visuospatial skills
Explicit	0.284 (p = 0.0134)	0.338 (p = 0.0030)	0.362 (p = 0.0014)	0.385 (p = 0.0006)	0.306 (p = 0.0077)
Logical	0.610 (p<0.0001)	0.502 (p<0.0001)	0.561 (p<0.0001)	0.447 (p = 0.0001)	0.593 (p<0.0001)
Pragmatic	0.628 (p<0.0001)	0.525 (p<0.0001)	0.562 (p<0.0001)	0.394 (p = 0.0005)	0.629 (p<0.0001)
Other	0.570 (p<0.0001)	0.523 (p<0.0001)	0.515 (p<0.0001)	0.384 (p = 0.0007)	0.564 (p<0.0001)
Distractor	0.384 (p = 0.0007)	0.325 (p = 0.0045)	0.429 (p = 0.0001)	0.363 (p = 0.0014)	0.396 (p = 0.0004)

Spearman rank-order correlation. Statistical significance was defined as p < 0.01.

The overall analysis of the whole sample showed that although all cognitive domains exhibited a certain degree of correlation with performance in all types of inferences, the strongest correlations (rho>0.5) were found between the domains of attention, memory, executive functions and visuospatial skills and *logical*, *pragmatic* and *other* questions. Language correlated only moderately with performance in all types of inferences.

Table 5 displays a more detailed analysis of the strongest correlations encountered in the overall sample, subdivided by diagnostic group.

**Table 5:** Correlations between performance on *logical*, *pragmatic* and *other* questions and cognitive domains by diagnostic group.

Type of question	Groups	Cognitive functions			
		Attention	Memory	Executive functions	Visuospatial skills
**Logical**	CG	0.365 (p = 0.074)	0.198 (p = 0.342)	0.255 (p = 0.218)	0.247 (p = 0.233)
	LHL	0.350 (p = 0.086)	0.035 (p = 0.866)	0.224 (p = 0.282)	0.352 (p = 0.084)
	RHL	0.358 (p = 0.079)	0.536 (p = 0.005)	0.387 (p = 0.056)	0.306 (p = 0.072)
**Pragmatic**	CG	0.245 (p = 0.238)	0.322 (p = 0.116)	0.048 (p = 0.818)	0.229 (p = 0.271)
	LHL	0.531 (p = 0.006)	0.197 (p = 0.344)	0.356 (p = 0.081)	0.473 (p = 0.001)
	RHL	0.090 (p = 0.667)	0.225 (p = 0.278)	0.110 (p = 0.600)	0.180 (p = 0.388)
**Other**	CG	0.099 (p = 0.635)	0.171 (p = 0.412)	0.053 (p = 0.800)	0.066 (p = 0.754)
	LHL	0.097 (p = 0.642)	0.314 (p = 0.126)	0.016 (p = 0.937)	0.087 (p = 0.678)
	RHL	0.548 (p = 0.004)	0.579 (p = 0.002)	0.417 (p = 0.038)	0.566 (p = 0.003)

Spearman’s rank-order correlation. Statistical significance was defined as p < 0.01.

In the RHL group, performance on *logical* questions was strongly correlated with memory alone; performance on *other* questions was strongly correlated with attention, memory, and visuospatial skills and moderately correlated with executive functions. In the LHL group, performance on *pragmatic* questions was strongly correlated with attention and moderately correlated with visuospatial skills.

## Discussion

The aim of this study was to compare the performance of patients with RHL and LHL, matched with cognitively healthy subjects, on a textual inference comprehension task. The possible influence of lesion site and the correlations between cognitive functions (attention, memory, language, executive functions and visuospatial skills) and performance on the inferential comprehension test were also examined.

### Performance of the groups on inferential comprehension

Regarding the overall analysis of the performance of the groups on the Implicit Management Test, the decreased total score of the RHL group confirmed that injuries to the right cerebral hemisphere result in heightened impairment of the ability to make inferences.

Possible causes for this difficulty are discussed in the literature. Classical explanations include impairment of the integration of previous world knowledge with new information [5], lack of semantic activation [6], and failure to inhibit the activation of irrelevant or inappropriate meanings in favor of the correct interpretation of the stimulus [7]. Blake [8] has verified that this failure is multifactorial and depends on the type of task and the strength of activation (predictability) of the inference, reinforcing the need to consider the nature of the stimulus for better comprehension of the alterations.

Thus, studies that compare the performance of subjects with RHL and LHL considering the type and complexity of the inferences required in the tasks can reveal new knowledge about the role of each brain hemisphere in inferential processing.

In this way, our results showed that lesions in both hemispheres impacted performance in all types of inferential tasks. However, the RHL group had greater difficulty than the LHL group in answering *logical*, *pragmatic* and *other* questions. Performance on *explicit* and *distractor* questions did not differ between the groups.

The performance differences among the groups may, in fact, be explained by the nature and complexity of the questions. According to Duchene-May-Carle [36], *logical* questions require logical interpretative strategies and deduction processes to provide the correct answer, which may require several steps of analysis. *Pragmatic* questions require the subject to carry out an induction process based on previous world knowledge, contextualization and consideration of discursive rules. *Other* questions demand even more complex cognitive strategies and require integration of formal reasoning within a contextualized situation, invoking a combination of logical and pragmatic competencies. In the *explicit* questions, the subject must understand paraphrases or make a literal translation of the utterance, and although the question may have been formulated differently, it is not necessary to add to the propositional content. *Distractor* questions evaluate the ability to ignore unnecessary or distractor information for comprehension, requiring the subject to inhibit an interpretative approach of the reading. Therefore, *logical*, *pragmatic* and *other* questions evaluate complex inferential reasoning, while *explicit* and *distractor* questions impose lower inferential demands.

Comparative studies have confirmed that lesions in either hemisphere can hinder the comprehension of different types of inferences. Hielscher (2004) [9] found that patients with RHL presented greater difficulty than patients with LHL in lexical decision tasks after reading texts that contained inferences with emotional background, which confirms the relationship between RH and emotional processing. Similarly, Saldert and Ahlsén (2007) [10] found that subjects with RHL failed in the processing of implicit inferences that required comprehension of the attitudes and motivations of the characters, whereas subjects with LHL presented difficulties in the inferences that required revision of more explicit content previously described in the text. Finally, Goel et al. (2007) [11] found that patients with RHL failed in drawing inferences from texts with incomplete information, while patients with LHL were impaired in trials with complete information.

Functional magnetic resonance imaging and visual paradigm studies in subjects without brain injury also have evidenced the activation of different patterns of hemispheric activation according to inferential demands. Sundermeier et al. (2005) [12] reported that the LH was involved in generating inferences for more familiar scenarios, while the RH was activated in less familiar scenarios. Virtue and van den Broek (2005) [13] investigated the activation of anaphoric inferences and showed that the LH had an advantage in the processing of consistent information, whereas the RH seemed to have an advantage in the processing of ambiguous concepts. Virtue et al. (2006b) [14] studied the generation of bridge and predictive inferences and found that both hemispheres were activated for inferences with stronger relationships, while there was greater activation of the RH than the LH for inferences with weaker relationships. Virtue and Moteska (2012) [15] described the predominance of the RH in processing inconsistent information, thus suggesting an influence of information coherence on the generation of inferences. Powers et al. (2012) [16] studied the understanding of narrative monologues and conversational dialogues, showing that subjects had greater activation of the RH during the comprehension of conversation (with predominance of more implicit processes) than narratives (with predominance of more explicit processes), demonstrating differences in semantic activation between the two hemispheres. Finally, Gouldthorp (2015) [17] demonstrated that during a lexical decision-making task to evaluate inferential sentence comprehension, the RH integrated the access to previous contextual information to maintain overall coherence (related to the general context), while the LH performed the “local” processing (related to the sentence itself) and maintained the activation of the most recent concept only.

Therefore, the contribution of the RH to inferential reasoning seems to vary, being more active in situations of more complex or more natural comprehension. While the LH is more involved in the literal, concrete and structural aspects of the linguistic system (phonology, morphology and syntax), the RH is predominantly related to discursive-pragmatic and nonliteral processing, which are responsible for the more contextualized use of language [18, 39–41]. In summary, the processing of inferences is considered a product of interhemispheric cooperation and requires the participation of all linguistic abilities. However, inference processing with high degrees of contextualization and complexity places higher demands on the RH than on the LH.

### Effect of lesion site on inferential comprehension

In the analysis of the effect of lesion site on the ability to understand inferences, our results showed only a trend-level difference in performance on *other* questions, where patients with right frontal lesions performed worse than patients with right posterior and left (anterior or posterior) lesions.

Functional neuroimaging studies show activation of bilateral areas during inferential processing, especially in frontal and temporal regions, such as the dorsolateral and medial prefrontal cortex, the inferior frontal gyrus, the cingulate cortex, and the anterior and medial parts of the superior temporal gyrus [18–25].

Some of these results highlight the importance of the frontal lobe during the generation of inferences [18, 22, 24]. While frontal areas are more related to the basic processing of language and management of cognitive resources, temporal areas are more important to the semantic integration at the level of sentences and text [20].

We believe that the absence of a relationship between performance and lesion site is due to our small sample size, which, combined with the mixed nature of the stimuli on the Implicit Management Test (conversation reports, descriptive narratives, and descriptions of problem situations, among others), may have prevented us from finding relevant differences.

### Influence of cognitive functions on inferential comprehension

In the overall sample, we found correlations between all cognitive domains and all types of inferences. However, the strongest correlations (rho> 0.5) occurred for *logical*, *pragmatic* and *other* questions and attention, memory, executive functions and visuospatial abilities, which confirms that these classes of questions are more complex and require higher cognitive demand than *explicit* and *distractor* questions.

Compared to controls, both the RHL and LHL groups exhibited increased dependence on attention and visuospatial skills for *pragmatic* and *other* questions.

Regarding attention, all types of attention (sustained, divided and selective) can impact the reading and understanding of complex materials. The comprehension of inferences during text reading may be impaired by deficits in initial decoding processes, which are dependent on saccadic movements and eye tracking [42], as well as by failures in subsequent stages, such as the selection of the stimuli of interest and difficulty in inhibiting irrelevant information [43]. Olkoniemi et al. [44] analyzed the eye movements of healthy adult individuals during the reading of figurative material (sarcasm and metaphor) and verified that, while the subjects were answering inferential questions, their eye movements became slower, which also evidences the intense relation between the comprehension processes for complex materials and working memory.

Visuospatial dysfunction may also affect the comprehension of inferences that require concepts of spatial analysis. *Pragmatic* questions, for example, sometimes required the subject to distinguish between right and left to provide the correct answer. McDonald [45] verified that the visuospatial functions actually contributed to pragmatic language impairments in individuals with RHL.

Compared to controls, RHL subjects exhibited increased dependence on memory for *logical* and *other* inferences.

Long-term memory enables the storage and retrieval of information that, when activated, contributes to reading comprehension through associations with previous knowledge [46]. The literature also highlights the intimate relationship between working memory and inferential processing. The higher the working memory capacity in terms of speed and storage, the more efficiently stimuli are decoded and the greater the availability of resources to perform operations of syntactic analysis, semantic integration of sentence components, and integration of sentences within the organization of the text [27]. Similarly, the studies of Virtue et al. [14, 19] on hemispheric activation have confirmed that the higher the inferential complexity, the greater the recruitment of working memory and the activation of the RH. Regarding the effects of brain injury, Saldert and Ahlsén [10] verified that the deficit of inferential comprehension during discourse in subjects with RHL was related mainly to sustained attention, while in the LHL group the deficit was correlated mainly with working memory.

With respect to the executive functions, failure in these abilities can affect the comprehension of inferences owing to rigidity of thought processes, predominance of more concrete answers, difficulty with abstract thinking, changes in behavioral regulation, and difficulty in inhibiting answers. Carriedo et al. [47] have verified the relationship between executive functions and comprehension of nonliteral material, such as metaphors.

Finally, the small influence of language on inferential comprehension was probably due to the intentional exclusion of patients with severe language or communication deficits prior to enrollment in the study.

## Conclusions

Lesions in both hemispheres may cause difficulties in the formation of inferences during reading. Patients with RHL presented more difficulties in the processing of *logical*, *pragmatic* and *other* inferences than LHL subjects or normal subjects did, which leads us to the interpretation that the right hemisphere contributes strongly to performance on tasks with higher cognitive demand. These findings confirm our first hypothesis.

We found a trend, although not statistically significant, for patients with right frontal lesions to perform worse than those with right posterior or left (anterior or posterior) lesions. These findings partially confirm our second hypothesis.

The ability to comprehend inferences was correlated with all extralinguistic cognitive domains, with especially strong influences from attention, memory, executive functions, and visuospatial functions for *logical* and *other* inferences in the RHL group. In the LHL, performance on *pragmatic* questions was correlated with attention and visuospatial skills. These findings partially confirm our third hypothesis, in that attention, memory and executive functions were correlated with inferential processing, but only for *logical*, *pragmatic*, and *other* questions. Even so, this correlation was asymmetrical for the RHL and LHL groups. However, we did not expect visuospatial abilities to be correlated significantly with inferential processing.

The major limitation of this study is the small sample size, which prevented us from performing a more comprehensive analysis of the impact of lesion site on each type of inferential processing. Opportunities for further studies on this subject include research using functional imaging to confirm the roles of the left and right hemispheres in relation to the nature and complexity of inferential reasoning.
